# *Plasmodium falciparum*: multifaceted resistance to artemisinins

**DOI:** 10.1186/s12936-016-1206-9

**Published:** 2016-03-09

**Authors:** Lucie Paloque, Arba P. Ramadani, Odile Mercereau-Puijalon, Jean-Michel Augereau, Françoise Benoit-Vical

**Affiliations:** CNRS, LCC (Laboratoire de Chimie de Coordination) UPR8241, 205 route de Narbonne, BP 44099, 31077 Toulouse Cedex 4, France; Université de Toulouse, UPS, INPT, 31077 Toulouse Cedex 4, France; Department of Pharmacology and Therapy, Faculty of Medicine, Universitas Gadjah Mada, Yogyakarta, Indonesia; Unité d’Immunologie Moléculaire des Parasites, Institut Pasteur, 75015 Paris, France

**Keywords:** Malaria, Artemisinin-based combination therapy, Resistance, PfK13, Quiescence

## Abstract

*Plasmodium falciparum* resistance to artemisinins, the most potent and fastest acting anti-malarials, threatens malaria elimination strategies. Artemisinin resistance is due to mutation of the PfK13 propeller domain and involves an unconventional mechanism based on a quiescence state leading to parasite recrudescence as soon as drug pressure is removed. The enhanced *P. falciparum* quiescence capacity of artemisinin-resistant parasites results from an increased ability to manage oxidative damage and an altered cell cycle gene regulation within a complex network involving the unfolded protein response, the PI3K/PI3P/AKT pathway, the PfPK4/eIF2α cascade and yet unidentified transcription factor(s), with minimal energetic requirements and fatty acid metabolism maintained in the mitochondrion and apicoplast. The detailed study of these mechanisms offers a way forward for identifying future intervention targets to fend off established artemisinin resistance.

**Electronic supplementary material:**

The online version of this article (doi:10.1186/s12936-016-1206-9) contains supplementary material, which is available to authorized users.

## Background

Artemisinin (ART) and its derivatives, introduced in 1980s, are the most potent and fastest acting anti-malarials, producing rapid clearance of parasitaemia and rapid resolution of symptoms. The discovery of artemisinin by Professor Youyou Tu has been rewarded the Nobel Prize for Physiology or Medicine 2015, recognizing that it has radically improved treatment against malaria [1]. *Plasmodium falciparum* artemisinin resistance was first detected in 2006 [2], after decreased clearance rates were observed in patients receiving an artemisinin therapy in clinical studies performed in 2006. It is nowadays widespread across southeast Asia [2, 3]. The delayed clearance of artemisinin-resistant infections exposes larger numbers of parasites to anti-malarial drugs, potentially driving selection to higher-grade artemisinin or to the partner drug. In Asia, treatment failures of artemisinin-based combination therapy (ACT) have recently emerged, including resistance to piperaquine [4]. This represents a serious threat for malaria eradication, widespread artemisinin resistance being predicted to cause in excess of 116,000 deaths annually, with medical costs and productivity losses evaluated as 146 million US$ and 385 million US$ per year, respectively [5]. The resistance to artemisinins is due to mutation of the PfK13 propeller domain endowing the parasites with an increased ability to enter a quiescent state. Piecemeal evidence associates artemisinin resistance with increased unfolded protein response (UPR), dysregulation of the pre-replication phase and the PI3K/PI3P/AKT pathway. Yet, an overall picture about how these modifications result in quiescence-associated artemisinin resistance is lacking. A conceptual framework for the cellular networks involved from oxidative stress to quiescence and parasite survival is proposed here, opening novel avenues for future research.

### Rapid overview of antiplasmodial drugs

Currently used anti-malarials belong to different chemical series and all *Plasmodium falciparum* stages can be targeted (asexual red blood cell stages, gametocytes, hepatic stages) by one or another anti-malarial. Each antiplasmodial drug series has its own specific mode(s) of action. In the food vacuole, haemozoin synthesis, corresponding to detoxification of the waste from haemoglobin by the parasite, is specially affected by quinolines [6] and artemisinins. Artemisinin and its derivatives are also responsible for alkylation of proteins and haem leading to oxidative damages [7]. At the mitochondrial level, atovaquone, targeting the cytochrome *bc*_*1*_ complex, inhibits the parasite electron transport chain and thus the dihydro-orotate dehydrogenase (DHODH) activity linked to the respiratory chain and involved in pyrimidine nucleotide biosynthesis [8]. In the cytosol, inhibition of dihydrofolate reductase (DHFR) by proguanil or pyrimethamine or of dihydropteroate synthase (DHPS) by sulfadoxine [9], blocks the biosynthesis of folate involved in DNA and RNA synthesis. Unfortunately, most anti-malarial drugs have lost their efficacy as resistance has emerged and spread.

The World Health Organization (WHO) defines resistance to anti-malarials as “the ability of *Plasmodium* to survive and/or multiply despite the administration and absorption of a medicine given in doses equal to -or higher than- those usually recommended but within the tolerance of the subject”, with the subsequent statement that “the form of the drug active against the parasite must be able to gain access to the parasite or the infected red blood cell for the duration of the time necessary for its normal action” [10]. For some anti-malarial drugs, resistance occurred very quickly after their introduction (Table 1).

**Table 1:** Date of introduction and first reports of anti-malarial drug resistance, resistance genes involved [33, 86, 87] and main mechanisms of resistance

Anti-malarial drug	Introduced	First reported resistance	Resistance genes	Main resistance mechanisms Refs	
Quinine	1632	1910	*Pfmdr1/other*	Disruption of drug accumulation inside the food vacuole by reduced propensity of the drug transporter PfMDR1 to bind to and transfer the anti-malarial	[88, 89]
Chloroquine	1945	1957	*Pfcrt/Pfmdr1*	Drug extrusion on from digestive vacuole by mutated drug transporter PfCRT due to higher lipophilicity and negativity of the transporter allowing ionized chloroquine efflux	[90–92]
Proguanil	1948	1949	*Pfdhfr*	Modification of the drug target: reduced inhibition of enzymatic activity by the drug	[9]
Sulfadoxine-pyrimethamine	1967	1967	*Pfdhps*-*Pfdhfr*		
Mefloquine	1977	1982	*Pfmdr1/other*	Reduction of parasite susceptibility to mefloquine by amplification of *Pfmdr1* copy number	[93]
Atovaquone	1996	1996	*Pfcytb*	Modification of the drug target by disruption of Cytochrome *bc1* complex	[8]
Artemisinins	1980s	2006	*Pfk13*	Quiescence	[31, 33]

Among all mechanisms of resistance described in various organisms, from bacteria, parasites, fungi to human cancer cells, two main mechanisms drive *Plasmodium* resistance to almost all anti-malarial drugs: (i) reduced drug availability at its site of action, essentially due to mutations in transporter genes; and, (ii) modification of the drug target by mutations in corresponding genes (Table 1). Artemisinin resistance results from a different cellular process, quiescence, which is detailed below. The quiescence-based cellular mechanisms comply with the WHO definition of *P. falciparum* drug resistance [10] (see also WHO global report on drug resistance 2010), as artemisinin-resistant parasites survival exposure to therapeutic, lethal concentrations of artemisinin derivatives.

### Recommendations for the use of dual and triple therapy

Parasite resistance to drug monotherapy prompted the WHO to recommend dual or triple therapy, which combines molecules with independent modes of action or distinct target enzymes. Drug combination is usually more effective and in the event of resistance to one component, the second one kills residual resistant parasites. However, some combination therapy, such as sulfadoxine-pyrimethamine plus chloroquine or amodiaquine, must be avoided due to the high levels of resistance established to these drugs already extensively used in monotherapy. For the same reason and because of the rapid acquisition of atovaquone resistance, atovaquone-proguanil is not recommended [2, 11, 12]. Clinical resistance to artemisinin was first detected in Cambodia in 2006 and reported in 2008 [13]. In 2009, Noedl et al. reported artemisinin resistance of uncomplicated falciparum malaria cases in Thailand and Cambodia and correlated delayed clearance with increased in vitro artemisinin IC_50_ values compared to the artemisinin-sensitive clone W2 [14]. Dondorp et al. [15] documented reduced in vivo susceptibility to artesunate in Pailin (western Cambodia) compared to Wang Pha (northwestern Thailand), characterized by a slow parasite clearance time (72–84 h in Pailin and 48–54 h in Wang Pha) and higher failure rates (recrudescence) after artesunate monotherapy (in Pailin 30 % of patients showed recrudescence compared to 10 % in Wang Pha). These altered clinical characteristics were not associated with reduced in vitro susceptibility, contrasting with observations by Noedl et al. [15]. Resistance to artesunate monotherapy first reported in western Cambodia and along the Thailand-Myanmar border, resulted in lengthening of the parasite clearance half-lives from 2.6 h in 2001 to 3.7 h in 2010 [16]. Artemisinin resistance is now also established in Myanmar, Vietnam, Lao PDR, and China [3].

In order to avoid the spread of *P.falciparum* resistance, monotherapy with artemisinin or its derivatives should be banned [17]. ACT has been recommended by the WHO as first-line treatment for uncomplicated malaria since 2001 and is nowadays widely used (Additional file 1). However, in December 2014 eight countries, mainly in Africa (Angola, Cabo Verde, Colombia, Equatorial Guinea, Gambia, Sao Tome and Principe, Somalia, Swaziland) still allowed artemisinin-based monotherapy [18].

Treatment failures with ACT have only been observed in Asia where, in addition to artemisinin resistance, parasites were also resistant to companion drug, e.g. mefloquine along the Thai-Myanmar border [19] or piperaquine in Cambodia [20–22]. In such cases, the ACT used is replaced by another one and the therapeutic efficacy of the replacement ACT must be monitored every 2 years as recommended by the WHO [2] for timely adaptation of the treatment policy. The threshold of 10 % cases with detectable parasites on day 3 after ACT treatment used by the WHO to define areas of artemisinin resistance [2] was established in Asia, a region of low/moderate transmission. In Africa, where transmission is usually much higher, artemisinin resistance has not yet been reported. However, a recent study suggested that a threshold of 5 % cases on day 3 parasite positivity is more suited to artemisinin resistance monitoring in Africa due to higher levels of acquired immunity against *Plasmodium* in African populations contributing to faster parasite clearance [23].

### Phenotypic and genotypic basis of artemisinin resistance

It is striking to note that from chloroquine resistance in 1957 to artemisinin resistance in 2006 [13], all anti-malarial drug resistance plaguing Asia was first reported at the Thai-Cambodian border and more especially in Pailin Province. Poor access to medicines, generating traffic of counterfeit drugs, with sub-clinical quantities, use of monotherapy, combined with an intense and extensive migrant labour system, are potential contributors [24–26]. No correlation was found between an increased mutation rate of the parasite genome and drug resistance in these regions, invalidating the hypothesis of the existence of a ‘hypermutator’ parasite [27]. Resistance in this region has a typical multi-resistance profile, with resistance mutations in *Pfcrt, Pfdhps, Pfdhfr, Pfmdr1* fixed in the parasite populations [28, 29].

Resistance to artemisinins was selected from such multi-resistant parasites circulating in western Cambodian provinces and was shown to be an inheritable genetic trait of the parasites [16, 30]. The mechanism driving artemisinin resistance was discovered using an experimental model, the F32-ART line -a highly artemisinin-resistant line- established in vitro after 5 years of exposure to escalating concentrations of artemisinin [31]. Artemisinin resistance of F32-ART is mediated by a resistance mechanism quite distinct from those previously described [15, 31–33] (Table 1), since these parasites score susceptible using the standard in vitro susceptibility assays [31, 33–35]. *Plasmodium* resistance to artemisinins is due to the enhanced number of young ring forms to enter into a quiescence state upon exposure to artemisinins, and quickly resume growth once the artemisinins are removed. This capacity is conferred by mutations of a gene called *Pfk13* [15, 31, 33]. This gene is now currently monitored to follow artemisinin resistance spread according to WHO recommendations [2, 18, 36].

### *Plasmodium falciparum* resistance to artemisinin is quiescence-based

The first hypothesis of artemisinin resistance based on a partial cytostatic effect of the drug was evoked thanks to a mathematical model in 2000 [37]. The classical definition of quiescence is ‘a reversible absence of proliferation’, i.e., a non-dividing cell which eventually restarts its cell cycle when conditions become appropriate. Various quiescent states may depend on the cell history; alternatively, quiescence can be the convergence of an adaptive process to cope with an adverse environment and an active preparation to efficiently resume proliferation [38]. The development cycle of *P*. *falciparum* presents normal arrest at the sporozoite stage in the mosquito salivary glands or at the gametocyte stage in red blood cells. In vitro, *P. falciparum* cell cycle progression can be strongly delayed at the trophozoite stage in response to isoleucine starvation [39]. A fraction of *P. vivax* and *Plasmodium ovale* sporozoites is able to stop its cell cycle during the hepatic phase to enter into a particular state, named hypnozoites, another form of dormant or quiescent parasites, and responsible for relapses, all under an epigenetic control of gene expression by histone modification enzymes [40].

#### Quiescence and artemisinin-resistant parasite stages

Data obtained with the highly artemisinin-resistant F32-ART line and *P. falciparum* isolates showed that artemisinin pressure induces developmental arrest of a sub-population of very young (0–3 h) ring stages which enter a quiescent state, while killing all other stages [31, 41]. Quiescence has also been correlated with K13-mediated artemisinin resistance in field isolates [33].

Quiescent parasites are difficult to identify under the microscope but staining mitochondrial activity with fluorescent dyes, unambiguously labels live quiescent cells in artemisinin-treated cultures [31, 42, 43]. Cell cycle arrest leads to overestimations of the parasite clearance time in patients [44], and a defect of H^3^-hypoxanthine incorporation in standard chemosensitivity assay leads to an overestimation of the effectiveness (IC_50_) of anti-malarials [31, 42]. As a result, classical susceptibility assays based on parasite proliferation are not suitable to differentiate parasites that are artemisinin-resistant from those that are artemisinin-sensitive. Recrudescence assays and ring stage survival assay (RSA_0–3h_) using highly synchronized *P. falciparum* parasites in culture, based on a 6-h dihydroartemisinin exposure in vitro, followed by culture in drug-free conditions until microscopic read-out at 72 h, are able to detect and quantify artemisinin resistance [31, 35, 41]. Quiescence takes place at a moment of the parasite cycle (young ring stage) where artemisinin is less efficient [43]. Indeed, artemisinin toxicity is potentiated by products of haemoglobin digestion inside the food vacuole, which begins in the mid-ring stage. Haemoglobin degradation products, such as haem and ferrous iron, react with artemisinin to cause oxidative stress and irreversible damages [45]. The low levels of haemoglobin endocytosis and therefore its absence or low digestion level in very young rings, could explain their reduced sensitivity. At this stage, which is approximately 14 h longer in resistant parasites than in wild-type parasites [46], a lower (moderate) oxidative stress is likely more manageable by the parasite, and particularly so by PfK13 mutants, while at the trophozoite stage, artemisinin-dependent damage exceeds the parasite’s response abilities [43]. The ring stage arrested development allows parasites to survive during the relatively short period of elevated artemisinin concentration (the elimination half-life in humans varies from one to 11 h depending on the derivative) [31, 41, 47]. Why only a small sub-set population (<1 %) of resistant parasites [42] is able to enter a quiescence stage despite their common genetic pattern remains to be elucidated.

#### Response of non-mutated PfK13 parasites to artemisinin exposure

It appears that all parasite lines are able to enter quiescence or dormancy-mediated processes [48]. In vitro, this is monitored by the capacity to proliferate after drug removal, i.e., to recrudesce. Artemisinin-resistance is characterized by a short time to recrudescence as demonstrated with the F32-ART (artemisinin-resistant) and F32-TEM (sensitive) sibling laboratory lines. After exposure to up to 18 µM of artemisinin, F32-TEM was able to resume growth and reach 5 % of parasitaemia 17 days after artemisinin removal compared to 10 days for F32-ART [31]. Other studies reported that several artemisinin-sensitive *P. falciparum* strains restarted their cell cycle from 4 to 25 days after the drug removal [48, 49]. Thus, some wild-type parasites are able to survive artemisinin exposure but artemisinin-resistant strains restart faster with more quiescent parasites and/or quicker awakening [31, 35].

### Involvement of the *Pfk13* locus in *Plasmodium falciparum* resistance to artemisinins

#### *Pfk13* polymorphism

Whole genome-sequencing of F32-ART and its sibling-sensitive line F32-TEM as well as of artemisinin-resistant *P. falciparum* isolates from Cambodia, allowed artemisinin resistance to be associated with mutations of the K13 protein (accession number PF3D7_1343700), belonging to the kelch super-family of proteins. All mutations found in the *Pfk13* gene and correlated with artemisinin resistance are non-synonymous, and are present after position 440 in the propeller domain (K13-propeller) [33, 36, 50]. Just one of these mutations is sufficient to confer artemisinin-resistance [50]. There is a strong genetic correlation in Asia between K13-propeller mutation and a slow parasite clearance time (half-life longer than 5 h) [3, 28, 33, 36, 51–53]. In Myanmar, among the K13 mutations observed, C580Y and M476I (the mutation acquired by F32-ART parasites) were associated with delayed clearance-time [54]. In Southern China, at the Myanmar border, the single F446I mutation predominates and is associated with delayed clearance [52]. The current picture is that artemisinin-resistant parasites circulating in different geographic areas of Southeast Asia stem from several independent emergences of unique mutations in the K13-propeller domain [28, 36, 53] where the parasite genetic background could play a role in the propensity of K13 mutants to emerge. Three prevalent K13-propeller mutations -C580Y, R539T and Y493H- were shown to correlate strongly with increased parasite clearance in vivo and with increased survival rates in the in vitro RSA_0–3h_ [33, 55, 56]. Gene editing showed that introduction of the wild-type allele into resistant parasites procured a sensitive, very low RSA_0–3h_ survival rate (0.3–0.7 %) whereas the parent isolates harbouring a K13 mutation (R539T, I543T or C580Y) displayed 40–49 % RSA_0–3h_ survival. Conversely C580Y introduced into wild-type artemisinin-sensitive Cambodian clinical isolates and reference lines conferred varying degrees of in vitro resistance, suggesting additional contribution from the genetic background [50]. Importantly, Asian K13 mutations are generally not found in Africa where numerous additional but rare *Pfk13* alleles were identified [36, 57–59]. An exception is the SNP A578S, observed in many African countries [58–60] as well as Bangladesh [51] and Thailand [36], however this mutation is not associated with artemisinin resistance [36].

Apart from one study in a limited number of African children with severe malaria [61], none of the K13 mutations has been associated with clinical artemisinin resistance for the time being, despite evidence that introducing the C580Y mutation generates artemisinin resistance in vitro in the NF54 parasite strain considered to be of African origin [62]. Genetic determinants of artemisinin resistance have been reviewed recently by Fairhurst [63]. A search of mutations in the *k13P. vivax* orthologue showed reduced polymorphism compared to *Pfk13* and the V552I mutation identified cannot be currently associated with any *P. vivax* drug resistance [64].

#### PfK13 activity

**Fig. 1:**
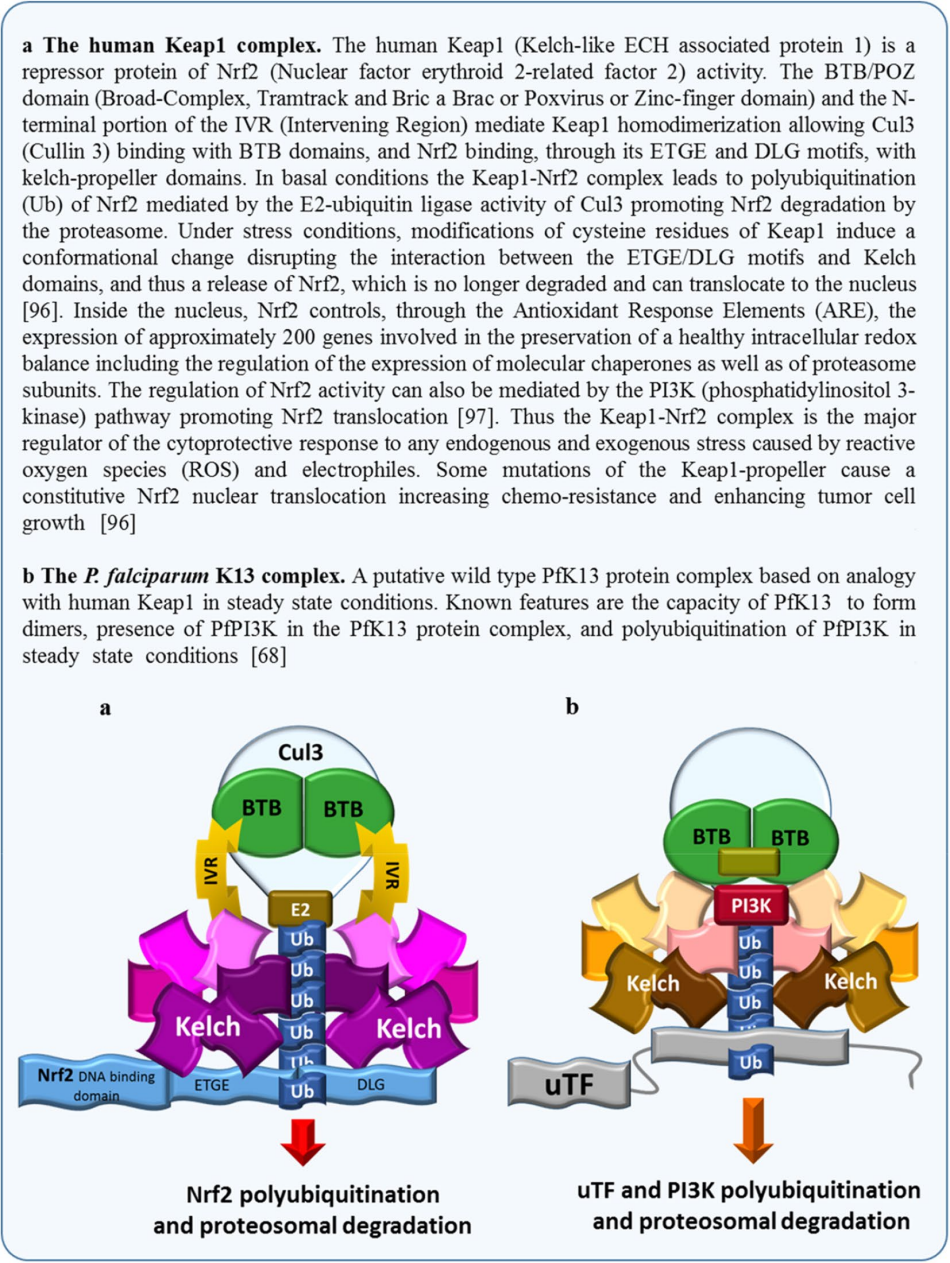
The Keap1 complex in human cells and a hypothetical PfK13 complex

The exact function of PfK13 in *Plasmodium* is not known yet, but analogies are possible with the human Keap1 protein function, in particular, in the cell response to oxidative stress (Fig. 1). PfK13 and Keap1 share homologies in the C-terminal BTB/POZ and the 6-kelch propeller domains [33, 65]. The presence of a BTB/POZ domain suggests that PfK13 could dimerize like Keap1. Formation of a dimer has been experimentally confirmed in the recently solved crystal structure of the PfK13 BTB/POZ propeller domain [66]. Based on Keap1 function, a hypothetical model could be that in steady state conditions, the wild-type K13-propeller domain binds to uTF (a putative, unidentified transcription factor functionally equivalent to human Nrf2) allowing its ubiquitination and proteosomal degradation. This ubiquitination could be mediated by one of the *Plasmodium* ubiquitin ligases but this has not yet been investigated. Importantly, the putative uTF transcription factor remains to be identified in the *Plasmodium* genome [67]. Recently, *P. falciparum* phosphatidylinositol 3 kinase (PfPI3K) was shown to undergo a PfK13-dependent ubiquitinylation. It could be immuno-precipitated in a complex with PfK13 [68] and has been included as a complex with PfK13 illustrated in Fig. 1, but direct binding of PfPI3K to PfK13 remains to be demonstrated [68].

### Biochemical consequences of a mutant K13 propeller

**Fig. 2:**
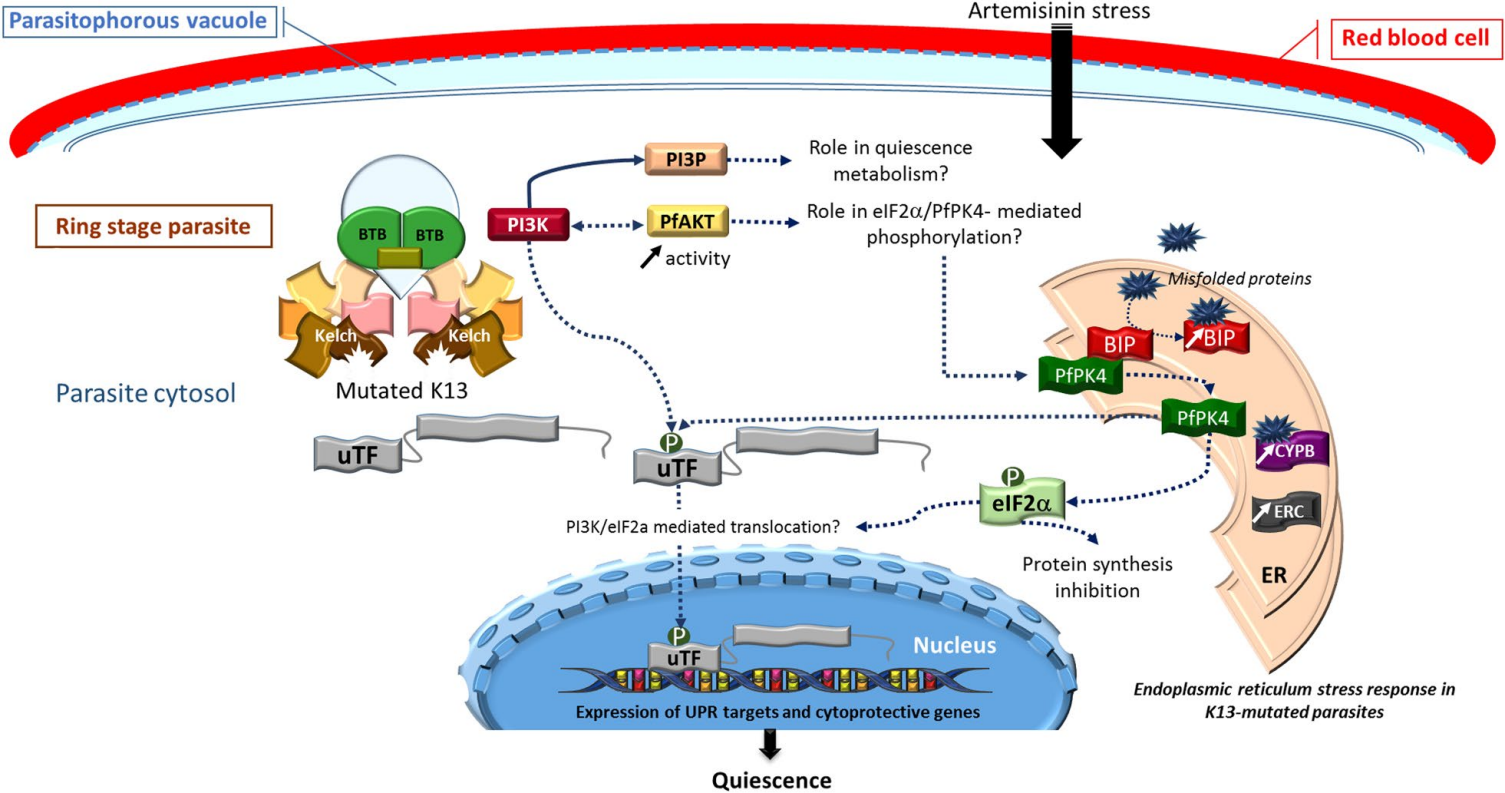
Mechanism proposed for PfK13-mediated resistance to artemisinins in K13 mutated *Plasmodium falciparum* at young ring stage. K13-propeller mutation prevents the fixation of uTF and PI3K to the Kelch domain and their ubiquitination, leading to enhanced concentration of PI3P. The artemisinin-induced oxidative stress is responsible for the accumulation of misfolded proteins in the *ER* endoplasmic reticulum. Misfolded proteins bind to BiP, the complex BiP-PfPK4 is dissociated and PfPK4 phosphorylates uTF and elF2α. elF2α phosphorylation should allow the translocation of uTF in the nucleus to regulate UPR targets and cytoprotective gene expression and also inhibit protein synthesis [75]. *BiP* immunoglobulin-binding protein, *BTB* broad-complex, tramtrack and Bric a Brac, *CYPB* cyclophilin B, *eIF2α* eukaryotic translation initiation factor 2α, *ERC* endoplasmic reticulum resident calcium binding protein, *PfPK4* *P. falciparum* protein kinase 4, *PI3K* phosphatidylinositol 3 kinase, *PI3P* phosphatidylinositol 3 phosphate, *uTF* unidentified transcription factor(s)

Based on Keap1 involvement in human lung cancer [69] and hypertension [70], it is possible to predict that K13-propeller mutations altering the propeller structure impair its biological function and interaction with partner proteins [33]. In particular, K13-propeller mutations would prevent fixation of uTF to the propeller domain, and as a consequence reduce the ubiquitinylation-dependent turnover, promoting translocation of uTF to the nucleus (Fig. 2).

In C580Y mutant parasites, PfPI3K was no longer ubiquitinylated, resulting in 1.5–twofold increased basal PI3P levels. This may reflect disrupted interactions between PfPI3K and PfK13 or disrupted interactions within the PfK13 associated protein complex (Fig. 2). Elevated basal phosphatidylinositol 3 phosphate (PI3P) levels appear central to artemisinin resistance, as K13 wild-type parasites became resistant upon elevation of PI3P levels induced by transgenic expression of human VPS34. Similarly transgenic elevation of PfAKT (also known as protein kinase B) in a wild-type parasite confers in vitro artemisinin resistance in connection with increased levels of PI3P by a feedback mechanism supposed [68]. As PfPI3K enzymatic activity is inhibited by artemisinins, the levels of PI3P likely drop rapidly upon exposure to artemisinins in sensitive parasites, whereas the PI3P level is already elevated in resistant parasites [68]. Additional downstream effectors likely come into play to orchestrate the quiescence response to artemisinin and withstand its toxicity.

**Fig. 3:**
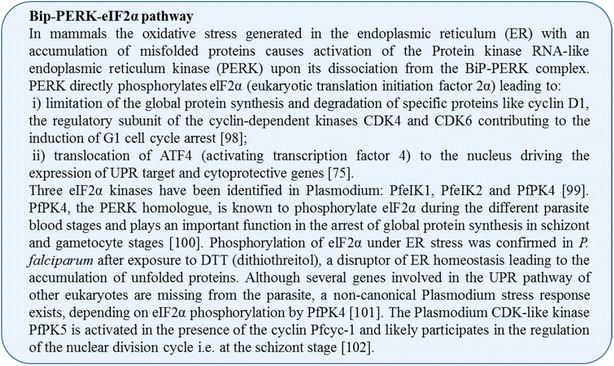
Bip-PERK-eIF2α pathway

PfK13 polymorphisms are also associated with modifications of endoplasmic reticulum homeostasis (Fig. 2). Indeed, transcriptome analysis of *P. falciparum* isolates revealed that artemisinin resistance is associated with increased expression of a network of molecular chaperones and major protein complexes belonging to the UPR pathway. In particular, immunoglobulin-binding protein (BiP) and cyclophilin B (CYPB), belonging to the *Plasmodium* reactive oxidative stress complex involved in protein folding and repair in the endoplasmic reticulum, are upregulated. Along the same line, the endoplasmic reticulum-resident calcium-binding protein (ERC) involved in endoplasmic reticulum Ca^2+^ homeostasis is also overexpressed [71, 72]. This upregulation of UPR in PfK13 mutants likely endows the parasite with increased ability to repair or degrade proteins damaged by alkylation and oxidation generated by artemisinin [73]. Based on homology with the human BiP-PERK-eIF2α pathway involved in the cell cycle arrest under stress conditions (Fig. 3) [74, 75], BiP bound to misfolded/altered proteins could dissociate from the PERK homologue, PfPK4, leading to its activation, and phosphorylation of eIF2α, which could trigger parasite cell cycle arrest via cyclin-dependent kinases, inhibition of protein synthesis, and the translocation of an unidentified transcription factor uTF into the nucleus. In mammalian cells, the PI3K/AKT pathway participates in UPR regulation and the activated AKT protein (also known as protein kinase B) seems to be required for Protein kinase RNA-like endoplasmic reticulum kinase (PERK)-mediated eIF2α phosphorylation [76]. This is reminiscent of the *Plasmodium* cell cycle slow down and activation of eIF2α kinases (PfeIK1 and PfeIK2) induced by amino acid starvation and observed in sporozoite latency inside mosquito salivary glands [39, 77]. Translocation of uTF could, in addition, be favoured by its phosphorylation by PfPK4 and also, like in humans, by PfPI3K, and could activate the expression of *Plasmodium* UPR and cytoprotective genes (Fig. 2).

This model does not exclude the existence of more than one uTF. Moreover, the PfPK4/PfPI3K downstream regulation cascades remain to be elucidated and experimentally demonstrated (Fig. 2). The involvement of the PfAKT pathway in artemisinin resistance, like PI3P involvement, still remains to be detailed. Moreover, the exact kinetics of events once the parasites are exposed to artemisinin oxidative and alkylating damage needs to be clarified, in particular as PI3K is inhibited by artemisinins [68] and should no longer contribute to refuelling PI3P levels.

Artemisinin resistance possibly includes a combination of the increased degradation of misfolded proteins/repair of damaged proteins especially those involved in the cell cycle progression and the ‘PfPK4/PfPI3K’ mediated response to the artemisinins’ oxidative stress. This cellular response of K13 mutant parasites to artemisinin stress could lead to a sub-set of ring stage parasites to enter into a quiescence state (Fig. 2). Why only a sub-set and a variable fraction of ring stages is involved needs to be understood. It may reflect a finely tuned balance between different cellular effectors that is unequally distributed between individual infected cells.

### Artemisinin-induced quiescence and *Plasmodium* metabolism

**Fig. 4:**
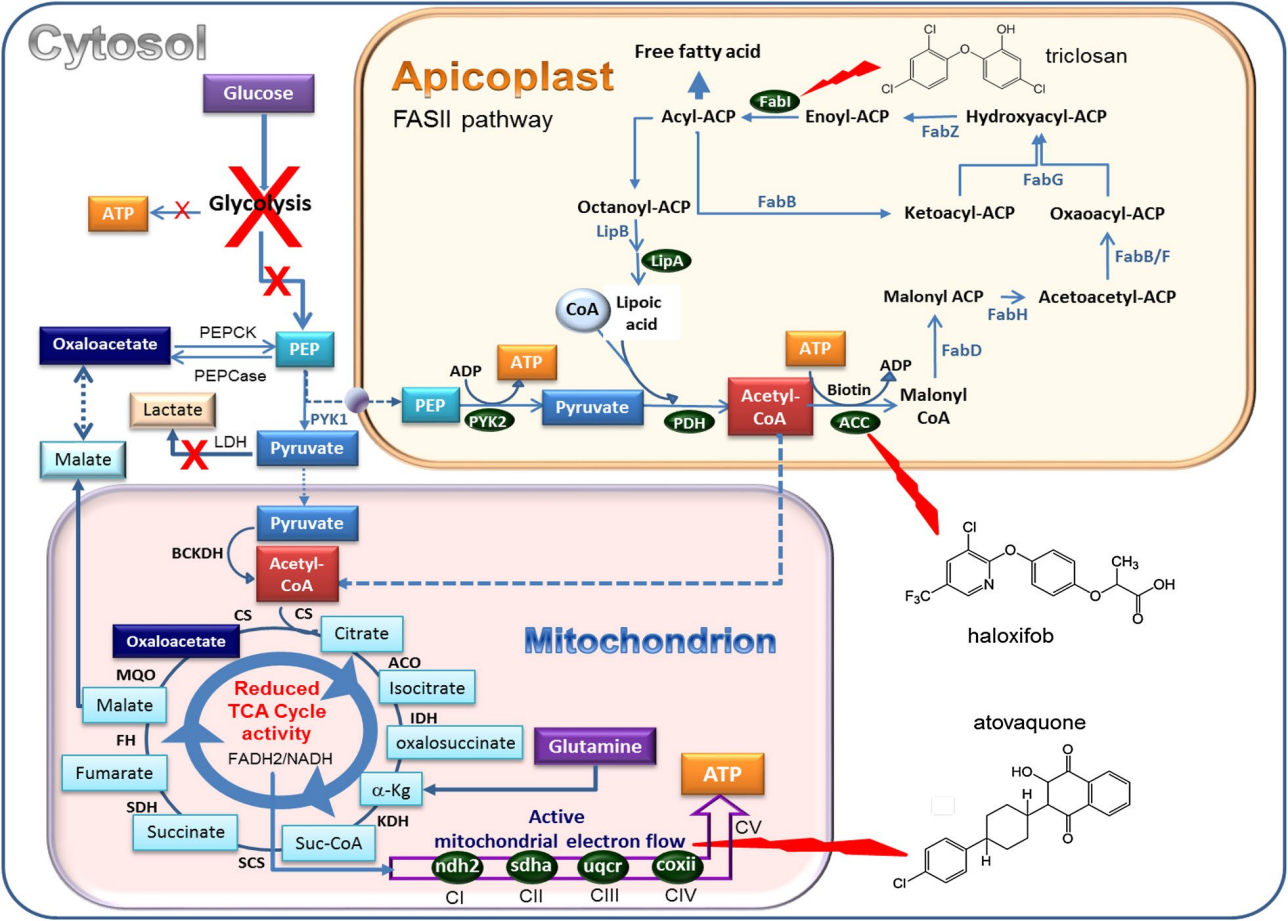
Synthetic model of *Plasmodium* quiescence metabolism. Quiescent *P. falciparum* parasites demonstrated an arrested glycolysis pathway leading to suspended production of ATP and phosphoenolpyruvate. Basal metabolism is maintained in quiescent parasites due to FASII metabolism in the apicoplast coupled with ATP production in the mitochondrion. Haloxifob, triclosan and atovaquone can disrupt these biochemical pathways [49, 95]. *Green* protein/enzyme with maintained expression in quiescent rings [49]. *ACC* acetyl-CoA carboxylase, *ACO* aconitase, *BCKDH* branched-chain keto acid dehydratase, *coxii* cytochrome c sub-unit II, *CS* citrate synthase, *FabI* enoyl-ACP reductase, *FabZ* β-hydroxyacyl-ACP dehydratase, *FabG* β-ketoacyl-ACP reductase, *FabB/F* β-ketoacyl-ACP synthase I/II, *FabH* β-ketoacyl-ACP synthase III, *FabD* malonyl-CoA:ACP transacylase, *FASII* fatty acid synthesis type II, *FH* fumarate hydratase, *IDH* isocitrate dehydrogenase, *KDH* α-ketoglutarate dehydrogenase, *LDH* lactate dehydrogenase, *LipA* lipoic acid synthase, *LipB* octanoyl-ACP protein transferase, *MQO* malate quinone oxidoreductase, *ndh2* NADH-ubiquinone oxidoreductase II, *PEP* phosphoenolpyruvate, *PEPCase* PEP carboxylase, *PEPCK* PEP carboxykinase, *PDH* pyruvate dehydrogenase, *PYK* pyruvate kinase, *SCS* succinyl-CoA synthase, *SDH* succinate dehydrogenase, *sdha* flavoprotein subunit of succinate dehydrogenase, *TCA* Tri carboxylic acid, *uqcr* iron sulfur sub-unit of ubiquinol Cytochrome c reductase [49, 83, 95]

Dihydroartemisinin (DHA)-induced quiescence in non-mutated PfK13 parasites is associated with suspended RNA, DNA and protein synthesis [49]. Folate metabolism, isoprenoid metabolism, lactate dehydrogenase activity and glycolysis, the main metabolic pathway producing adenosine triphosphate (ATP) and phosphoenolpyruvate (PEP) in *Plasmodium*, are severely down-regulated [49]. But, even though the metabolism of quiescent parasites is largely down-phased, some pathways remain active and require another source of ATP and PEP, depending on pyruvate production by the apicoplast and on mitochondrial activity (Fig. 4) [49, 78]. In DHA-induced quiescent rings, among the genes that remain actively transcribed, are genes coding for enzymes of the pyruvate metabolism (pyruvate kinase 2, pyruvate dehydrogenase E1 beta subunit), the fatty acid metabolism by FASII -fatty acid synthesis type II- pathway (biotin acetyl-CoA carboxylase, enoyl-acyl carrier reductase/FabI) and for the lipoic acid metabolism (lipoic acid synthase) [49]. The mitochondrial tricarboxylic acid cycle is down-regulated but remains active and genes encoding the proteins of the electron transport chain also remain transcribed at a normal level (cytochrome c subunit II, NADH-ubiquinone oxidoreductase II, flavoprotein sub-unit of succinate dehydrogenase and ubiquinol cytochrome c reductase iron sulfur sub-unit) [49]. This was confirmed by rhodamine staining of parasite after DHA treatment: only rhodamine-positive parasites resumed growth [42]. In stress conditions, depletion of host-derived fatty acids induces an upregulation of the *Plasmodium* FASII pathway [79]. PI3P is present in the apicoplast membrane and can play a role in exchanges of protein and likely lipids [80–82] with the adjacent mitochondrion. The elevated production of PI3P in resistant parasites [68] could play a role in the maintenance of this minimal energetic metabolism based on mitochondrial and apicoplast activity and also found in quiescent resistant parasites [49]. Indeed these parasites cannot survive and resume growth in the presence of atovaquone, an inhibitor of the mitochondrial activity, and the use of haloxyfob, an acetyl-CoA carboxylase inhibitor, as well as triclosan, an inhibitor of FabI delays the recovery of DHA-induced quiescent parasites [35, 42, 49, 83, 84], demonstrating that this minimum active metabolism during the induced quiescence state by artemisinins is essential for the survival and the recovery of the parasites from dormancy.

Thus, the quiescent state induced at the young ring stage by artemisinin in sensitive and resistant parasites likely seems to involve the same cellular mechanisms, but the PfK13 mutation shifts the intra-population distribution so as to allow more parasites to become quiescent in the case of resistant parasites, and to quickly resume growth after the drug removal in vitro or drug elimination in patients.

## Conclusions

It is increasingly clear that the *P. falciparum* resistance to artemisinin and its derivatives is not due to efflux modulation or target modifications as described for other anti-malarials, but is based on increased capacity of PfK13-mutant parasites to manage oxidative damage thanks to greater UPR mobilization. The over-expression of UPR target genes should impact three key points: (an) unidentified transcription factor(s) (uTF) regulating transcription of UPR/oxidative response genes, the PI3 K/PI3P/AKT pathway activity and the PfPK4/eIF2α cascade. This would allow parasite entrance into a quiescence state with minimal energy metabolism provided by the apicoplast and the mitochondrion, maintained by alternative tricarboxylic acid cycle and FASII metabolism, until drug removal/excretion when parasites can resume growth. Recently, the artemisinin-resistant F32-ART line, selected by long-term drug pressure with solely artemisinin, was shown to display an extended age range of stages surviving artemisinin treatment extending to older ring stages (13–16 h) and even trophozoite stages. These parasites were also able to survive lethal doses of diverse classes of anti-malarial drugs, including molecules used as partners in currently recommended ACT, in the absence of the ‘classical’ mutations of the target genes for these drugs. Thus, long-term in vitro artemisinin exposure selects a novel multidrug tolerant phenotype, which could represent a major threat to anti-malarial drug policy in the field [35]. This threat is exacerbated by the fact that artemisinin-resistant parasites seem able to infect and be transmitted by a large panel of *Anopheles* species, including the major African species *Anopheles gambiae* [85]. This is yet a another reason to urgently clarify the cellular network of ART resistance in order to identify new therapeutic modalities (to protect the molecules currently used), and/or novel drug development, in order to avoid entrance into a quiescent state, target quiescent parasites and promote restart of the cell cycle, rescuing drug susceptibility of parasites.

## Additional file

10.1186/s12936-016-1206-9 ACT recommended by WHO and the newly approved combination DHA-piperaquine.
