# Supplementary figures and images for: Plasmodium falciparum: multifaceted resistance to artemisinins
Source: Malar J. 2016 Mar 9;15:149. doi: 10.1186/s12936-016-1206-9 (PMC4784301; doi:10.1186/s12936-016-1206-9)

**Additional file 1 ACT recommended by WHO and the newly approved combination DHA-piperaquine** **[2,18,94]**


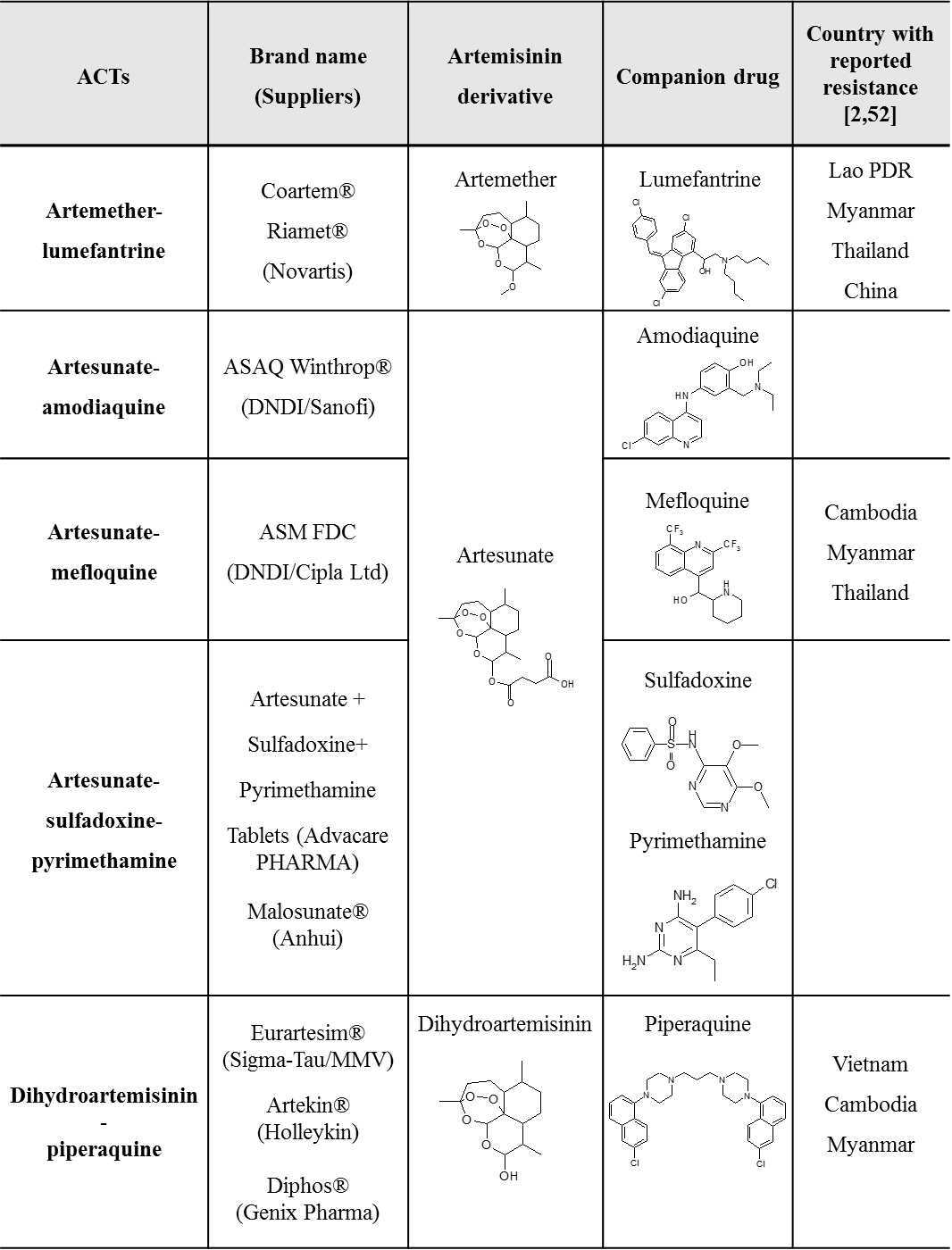

Supplement: Supplementary file 1 — 10.1186/s12936-016-1206-9 ACT recommended by WHO and the newly approved combination DHA-piperaquine. [file 12936_2016_1206_MOESM1_ESM.docx]
